# Educational

**Published:** 1871-06

**Authors:** C. Ellis

**Affiliations:** Dean, 114 Boylston Street, Boston


### EDUCATIONAL.

To show that the Ohio College of Dental Surgery no longer stands alone in the plan of its curriculum of study, we give below the announcement of the Medical department of Harvard University. This announcement is so gratifying that we can not refrain from copying it in full.

Now either Harvard University is very much mistaken and blundering terribly in this matter, or the Ohio College of Dental Surgery is on the right track It has seemed very strange to us for the last few years that almost all medical and dental teachers should recognize and freely admit that the plan of instruction usually employed is very imperfect and inefficient, and yet no effort be made at all for its improvement, either in the prior attainments or in the curriculum of study. The truth is that there exists such degrading competition in the medical and Dental schools throughout our country, with few exceptions, that the results of their labors is the casting upon society a host of incompetents, who assume with the most perfect assurance the duties of one of the most responsible occupations of life. Such a competition as involves the letting down the standard of requirements to a most debasing level, degrading all concerned; demoralizing both teachers and students.

Our colleges should have a door of entrance through which no one not properly prepared could enter, and an exit door at the top which none should be permitted to pass who had not properly and squarely mounted every step to it. Then and not till then will medicine and its specialties receive that regard and appreciation which the duties and responsibilities seem to require.

T.

HARVARD UNIVERSITY, MEDICAL DEPARTMENT,
BOSTON, MASS., 1871–72.

### CHANGES IN THE PLAN OF STUDY AND THE REQUISITES FOR A DEGREE.

The regular course of study for persons who begin their medical education at this school, will occupy three full years. The year will begin on the Thursday following the last Wednesday in September, and end on the last Wednesday in June, and will be divided into two equal terms. The instruction will be given by lectures, recitations and practical exercises, throughout the year. The general subjects of the regular course of study are:

*For the first year*—Anatomy, Physiology and general Chemistry.

*For the second year*—Medical Chemistry, Materia Medica, Pathological Anatomy, Theory and Practice of Medicine, Clinical Medicine, Surgery and Clinical Surgery.

*For the third year*—Pathological Anatomy, Therapeutics, Obstetrics, Theory and Practice of Medicine, Clinical Medicine, Surgery and Clinical Surgery.

No student will receive his degree until he has passed a satisfactory examination in all the above-mentioned subjects. Examinations in all these subjects will be held at the beginning, middle and end of each year.

Students who take the regular course of the school will be divided into three classes according to their time of study and proficiency. Students may be admitted to advanced standing in the regular course; but all persons who apply for admission into the second or third year's class must pass an examination in the branches already pursued by the class to which they seek admission. Students who fail in any subject at one examination may be examined again at the next examination. The regular examinations will be held in the following order:

At the end of the first year—Anatomy, Physiology and Chemistry.

At the end of the second year—Medical Chemistry, Materia Medica, and Pathological Anatomy.

At the end of the third year—Therapeutics, Obstetrics, Theory and Practice of Medicine, Clinical Medicine, Surgery and Clinical Surgery.

Students who begin their professional studies elsewhere may be admitted to the school and become candidates for a degree without joining the regular classes; such students may take up the subjects which they have not previously studied, in such order as may be thought best, passing the examinations at the beginning, middle and end of each year.

Students who do not intend to offer themselves for a degree, may join the school for one term or more, and pay for instruction in such subjects as they may select. Such students will be furnished, without examination, with certificates of attendance.

Requirements for a Degree.—Every candidate must be twenty-one years of age; must have studied medicine three full years, have spent at least one continuous year at this school, have passed the required examinations, and have presented a thesis.

Fees.—For matriculation, $5; for the year, $200; for either term, $120; for graduation, $30; for courses in single subjects, according to the detailed announcement.

The plan will go into operation on Sept. 28, 1871, but the changes above described will not affect students who have already entered the school, unless by their choice.

For further information, address

Dr. C. Ellis, *Dean*,
114 Boylston Street, Boston.

